# Interventions and symptom relief in hospital palliative cancer care: results from a prospective longitudinal study

**DOI:** 10.1007/s00520-021-06248-z

**Published:** 2021-05-03

**Authors:** Morten Thronæs, Erik Torbjørn Løhre, Anne Kvikstad, Elisabeth Brenne, Robin Norvaag, Kathrine Otelie Aalberg, Martine Kjølberg Moen, Gunnhild Jakobsen, Pål Klepstad, Arne Solberg, Tora Skeidsvoll Solheim

**Affiliations:** 1 Department of Clinical and Molecular Medicine, Faculty of Medicine and Health Sciences, NTNU - Norwegian University of Science and Technology, Trondheim, Norway; 2 Cancer Clinic, St Olavs University Hospital, Trondheim, Norway; 3 Department of Anaesthesiology and Intensive Care Medicine, St Olavs University Hospital, Trondheim, Norway; 4 Department of Circulation and Medical Imaging, Faculty of Medicine and Health Sciences, NTNU - Norwegian University of Science and Technology, Trondheim, Norway

**Keywords:** Cancer, Symptoms, Acute palliative care unit (APCU), Palliative, Integration, Symptom development

## Abstract

**Purpose:**

To study the use of interventions and symptom relief for adult patients with incurable cancer admitted to an acute palliative care unit providing integrated oncology and palliative care services.

**Methods:**

All admissions during 1 year were assessed. The use of interventions was evaluated for all hospitalizations. Patients with assessments for worst and average pain intensity, tiredness, drowsiness, nausea, appetite, dyspnea, depression, anxiety, well-being, constipation, and sleep were evaluated for symptom development during hospitalization. Descriptive statistics was applied for the use of interventions and the paired sample *t*-test to compare symptom intensities (SIs).

**Results:**

For 451 admissions, mean hospital length of stay was 7.0 days and mean patient age 69 years. More than one-third received systemic cancer therapy. Diagnostic imaging was performed in 66% of the hospitalizations, intravenous rehydration in 45%, 37% received antibiotics, and 39% were attended by the multidisciplinary team. At admission and at discharge, respectively, 55% and 44% received oral opioids and 27% and 45% subcutaneous opioids. For the majority, opioid dose was adjusted during hospitalization. Symptom registrations were available for 180 patients. Tiredness yielded the highest mean SI score (5.6, NRS 0–10) at admission and nausea the lowest (2.2). Significant reductions during hospitalization were reported for all assessed SIs (*p* ≤ 0.01). Patients receiving systemic cancer therapy reported symptom relief similar to those not on systemic cancer therapy.

**Conclusion:**

Clinical practice and symptom relief during hospitalization were described. Symptom improvements were similar for oncological and palliative care patients.

## Introduction

Cancer patients treated with palliative intent suffer from a diversity of symptoms [1–3]. The symptom burden remains high over time, both on a population level and throughout the disease trajectory [4–7]. Suboptimal symptom assessment and management are major contributors to inadequate symptom control [8]. The relevant and ongoing attention to effectiveness of healthcare services also warrants a focus on the interventions used to achieve symptom improvement [9–11].

Systematic symptom assessment is pivotal in palliative care and may improve survival [12, 13]. The patient perspective is an important element of cancer care, as the healthcare providers tend to underestimate the patient’s symptom burden [12, 14, 15]. Patient-reported outcome measure (PROM) is an umbrella term covering the patient’s perspective on physical and psychological well-being, including symptom severity, symptom impact, and treatment effects [12]. Assessment tools reporting PROMs are recommended and a multitude of symptom assessment tools exist [12, 16–18]. Although many studies report results based on systematic symptom assessment, publications on the use of interventions and overall symptom development during hospitalization in palliative care units are fewer and less comprehensive [19–21].

Integration of oncology and palliative care implies earlier referral to palliative care programs and palliative care units [12]. This approach may enhance symptom control and family satisfaction, improve survival, and represent a potential for better utilization of healthcare resources [12]. Acute palliative care units (APCUs) facilitating early integration of oncology and palliative care are endorsed by the European Society for Medical Oncology (ESMO) [22–24]. Rapid symptom relief, increased quality of life, and beneficial use of interventions are essential goals for the APCU stay [12, 25]. However, the optimal content of palliative care integrated into oncology is not established [12].

A 1-year healthcare improvement study was conducted in an ESMO Designated Centre of Integrated Oncology and Palliative Care, aiming to answer the following research questions:

1. Which interventions are used during hospitalization at the APCU?
2. How does patient-reported symptom intensity (SI) change from admission to discharge during hospitalization at the APCU?

## Methods

### Design

A prospective longitudinal study was conducted among inpatients at an APCU in a tertiary cancer clinic. All hospitalized patients admitted between January 15, 2019 and January 15, 2020 were assessed. With a dedicated organizational focus on systematic symptom assessment, clinical practice based on commonly accepted palliative care principles was carried out as usual [12].

### Organization of the APCU

The APCU comprises a 12-bed ward and an outpatient clinic at the Cancer Clinic, St. Olavs Hospital, Trondheim University Hospital, Norway. The Cancer Clinic is an ESMO designated Centre of Integrated Oncology and Palliative Care. The senior consultants at the APCU are oncologists with specialized training in palliative care and attend the Cancer Clinic’s common day-to-day activities, such as joint meetings and internal teaching. A residency in oncology includes at least 6 months of compulsory service at the APCU, where the majority of the nurses are trained in both oncology and palliative care.

The multidisciplinary team (MDT) at the APCU consists, in addition to physicians and nurses, of physiotherapists, occupational therapists, chaplains, social workers, and a clinical dietitian. Patients are followed up by specific professions or by the entire MDT.

### Patients

Adult patients with incurable cancer are referred to the APCU. Patients with hematological, gynecological, and pulmonary malignancies, and receiving treatment at their respective university hospital departments, are only referred when in need of neuraxial pain management. Patients on systemic cancer therapy and in need of palliative care are included in an integrated care pathway, and follow-up is a shared responsibility of the treating oncologist and the palliative care team. The oncologist is responsible for the tumor-directed treatment, while the palliative care physician is in charge of the symptom management. Joint consultations are encouraged and the patient perspective is paramount in the decision-making process regarding further treatment plans. However, the bulk of the patients are included in a “palliative care pathway” and solely treated by the palliative care team.

### Assessments and data collection

**Fig. 1:**
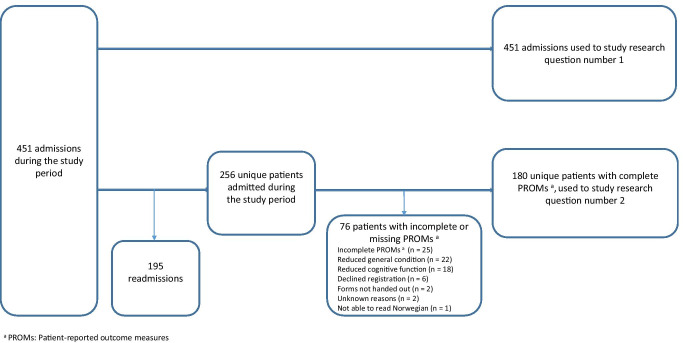
Patient inclusion and exclusion.

For the study purpose, the term “intervention” included diagnostic imaging, medical treatments, therapeutic and interventional radiology, surgery, and multidisciplinary follow-up. All consecutive admissions were assessed to describe the use of interventions during hospitalization. For the evaluation of symptom development, only unique patients with symptom registrations at admission and at discharge were included (Fig. 1).

The patients reported average SI past 24 h on the 11-point numeric rating scale (NRS 0–10) [26]. Daily PROMs were recorded on a paper-based form, and included the symptoms pain, tiredness, drowsiness, nausea, appetite, shortness of breath, depression, anxiety, well-being, constipation, and sleep [27]. In addition, worst pain intensity the past 24 h was assessed (NRS 0–10) [28, 29].

Physician-reported patient information included gender, age, cancer diagnosis, metastatic status, and medical comorbidity. Additionally, information on care pathway, interventions, and place of care after discharge was recorded.

### Statistical analysis

Descriptive statistics was applied for demographics, clinical data, and the use of interventions.

NRS = 0 indicated no symptoms, whereas mild SI was defined as scores of 1–3, and moderate to severe SI defined as scores of ≥ 4 (NRS 0–10) [26]. An absolute NRS reduction ≥ 1 during the hospital stay was considered a clinically important difference [30].

For all symptoms assessed, the paired sample *t*-test was used to compare SI at admission and at discharge.

For patients admitted more than once, only the first admission to the APCU was included in the analyses of symptom development, due to considerations on independent variables. Single imputations with last value carried forward were performed for patients with missing data at discharge.

The difference in symptom burden at admission and at discharge for patients included in the “integrated care pathway” and the “palliative care pathway,” respectively, was compared using the independent samples *t*-test.

Normal distribution was verified by visual inspection of Q-Q plots. A two-sided *p*-value < 0.05 was considered statistically significant.

### Ethics

The Regional Committee for Medical Research Ethics, Health Region Central Norway (REK) (2018/925/REK midt) defined the project as healthcare improvement, without the need for explicit informed consent from the patients.

## Results

Four hundred fifty-one admissions were registered during the 1-year study period, of which 302 (67%) were emergency admissions. Elective admissions and referrals from other hospital departments were equally distributed among the remaining. Mean hospital length of stay was 7.0 days (Table 1). Two hundred sixty (58%) of the patients were discharged to home care, 122 (27%) to nursing homes, and 57 (13%) died during hospitalization. Only a small fraction of the patients was discharged to other hospitals. One hundred ninety-five (43%) of the 451 hospitalizations were readmissions.

**Table 1:** Patient characteristics. All admissions

	Sample	Percentage
Age, years (standard deviation)	68.9 (13.1)	
Gender		
Male	272	60.3%
Female	179	39.7%
Marital status		
Living alone	163	36.1%
Married or cohabitant	278	61.6%
Missing	10	2.2%
Cancer diagnosis		
Gastrointestinal	196	43.5%
Urological	103	22.8%
Breast	48	10.6%
Lung	8	1.8%
Head/neck	36	8.0%
Others	59	13.1%
Missing	1	0.2%
Metastases		
Yes	397	88.0%
No	54	12.0%
Comorbidity ^a^		
Yes	316	70.1%
No	135	29.9%
Trajectory		
Palliative care pathway	265	58.8
Integrated care pathway	170	37.7
Other hospital care pathways	15	3.3
Missing	1	0.2
Mean hospital length of stay, days (range)	7.0 (1–34)	

^a^Comorbidity = cardiovascular disease, diabetes, renal failure, musculoskeletal disease, psychological illness, chronic obstructive pulmonary disease, liver disease, others

Baseline patient characteristics are described in Table 1. Mean age was 69 years and 60% were males. Gastrointestinal, urological, and breast cancer were the most frequent cancer diagnoses. Most patients were either married or cohabitant, and the vast majority had metastases and comorbidities. More than one-third of the patients were included in the “integrated care pathway”.

### Interventions

Diagnostic and therapeutic interventions (other than medical adjustments) during the 451 hospitalizations are displayed in Table 2. Diagnostic imaging was performed in 66% of the hospital stays. Computer tomography and plain X-rays were the most commonly used modalities. Intravenous rehydration was administered to 45% of the admitted patients, while 37% and 32% received antibiotics and any medical nutrition therapy, respectively. The MDT was involved in the follow-up of 39% of the patients. Most frequently, the physiotherapist was involved, followed by the occupational therapist, social worker, chaplain, and clinical dietitian.

**Table 2:** Interventions during hospitalization. All admissions

	Sample	Percentage^a^
Diagnostic imaging		
No	155	34.4
Yes ^b^	296	65.6
Computer tomography	165	36.6
Plain X-ray	156	34.6
Magnetic resonance imaging	55	12.2
Ultrasound	49	10.9
Scintigraphy	2	0.2
Rehydration	203	45.0
Antibiotic treatment	168	37.3
Medical nutrition therapy	146	32.4
Blood transfusion	76	16.9
Radiation therapy	53	11.8
Radiological interventions ^c^	48	10.6
Surgery	17	3.8
Follow-up by the multidisciplinary team		
No	275	61.0
Yes ^d^	176	39.0
Physiotherapist	121	26.8
Occupational therapist	62	13.7
Social worker	38	8.4
Chaplain	22	4.9
Clinical dietitian	15	3.3

^a^Percentage of all 451 admissions

^b^Some patients had multiple examinations

^c^E.g., stenting and drains

^d^Some patients were followed up by more than one member of the multidisciplinary team

Medical adjustments are reported in Table 3. At admission, 55% of the patients received oral opioids and 27% subcutaneous opioids. At discharge, the corresponding numbers were 44% and 45%, respectively. Ten percent of the patients did not receive opioids at admission. Dosing was adjusted for 68% of the patients using opioids and represented the most frequent medication adjustment. At admission, 69% of the patients received laxatives, 54% corticosteroids, and 53% antiemetics. At discharge, the corresponding numbers were 70%, 63%, and 59%, respectively.

**Table 3:** Medications at admission, at discharge, and dose adjustments. All admissions

	Medication	Patients using drug at admission *n* (%)	Patients using drug at discharge *n* (%)	Patients with dose adjustments during hospitalization ^a^ *n* (%)
Oral pain medications	Paracetamol	265 (58.8)	254 (56.3)	62 (13.7)
	NSAIDs ^b^	8 (1.8)	10 (2.2)	5 (1.1)
	Codeine and tramadol	23 (5.1)	14 (3.1)	14 (3.1)
	Morphine	109 (24.2)	99 (22.0)	88 (19.5)
	Oxycodone	113 (25.1)	83 (18.4)	72 (16.0)
	Methadone	3 (0.7)	3 (0.7)	1 (0.2)
	Adjuvant pain medications ^c^	52 (11.5)	50 (11.1)	23 (5.1)
Patch	Fentanyl	10 (2.2)	9 (2.0)	4 (0.9)
Subcutaneous pain medications	Morphine	83 (18.4)	144 (31.9)	126 (27.9)
	Oxycodone	32 (7.1)	48 (10.6)	48 (10.6)
	Hydromorphone	8 (1.8)	12 (2.7)	9 (2.0)
Miscellaneous medications	Antidepressants	76 (16.9)	76 (16.9)	21 (4.7)
	Anxiolytics	168 (37.3)	221 (49.0)	104 (23.1)
	Corticosteroids	244 (54.1)	283 (62.7)	170 (37.7)
	Laxatives	312 (69.2)	315 (69.8)	139 (30.8)
	Antiemetics	238 (52.8)	265 (58.8)	99 (22.0)
	Hypnotics	130 (28.8)	140 (31.0)	30 (6.7)

^a^Number of patients with dose adjustments was calculated for each drug/type of drug. Adjustments include drug initiation, dose increments, dose reductions, and drug discontinuation

^b^*NSAIDS* nonsteroidal anti-inflammatory drugs

^c^E.g., anticonvulsants or antidepressants used as pain medication

### Symptom development

PROMs at first admission and at first discharge were available for 180 unique patients (Fig. 1, Table 4). At admission, patient-reported tiredness yielded highest mean SI score (5.6, NRS 0–10), followed by worst pain and drowsiness (both 5.2), whereas the lowest mean SI was reported for nausea (2.2). Statistically significant reductions were reported for all assessed SIs during the hospital stay (*p* ≤ 0.01), and clinically important reductions (SI reduction ≥ 1, NRS 0–10) were reported for worst pain, tiredness, constipation, average pain, well-being, drowsiness, and appetite. For the subgroup of patients with moderate to severe SIs (NRS 4–10) at admission, both statistically significant and clinically important reductions in SI were reported for all assessed symptoms (Table 5).

**Table 4:** Symptom intensity and symptom development during hospitalization. Unique patients with complete data

Symptom	Sample	Mean NRS ^a^ score			SD ^b^	*p*-value ^c^
		Admission	Discharge	Difference ^d^		
Average pain	178	3.84	2.75	1.08	2.44	< 0.01
Tiredness	178	5.60	4.34	1.26	2.27	< 0.01
Drowsiness	176	5.18	4.11	1.07	2.50	< 0.01
Nausea	180	2.15	1.34	0.81	2.16	< 0.01
Appetite	171	4.50	3.50	1.00	2.73	< 0.01
Shortness of breath	174	3.26	2.37	0.89	2.15	< 0.01
Depression	175	3.48	2.89	0.59	2.28	< 0.01
Anxiety	176	2.95	2.25	0.70	2.18	< 0.01
Well-being	162	4.51	3.44	1.07	2.57	< 0.01
Constipation	167	3.10	1.91	1.19	3.08	< 0.01
Sleep	176	4.03	3.22	0.82	3.10	< 0.01
Worst pain	161	5.20	3.66	1.55	2.84	< 0.01

^a^*NRS* numeric rating scale

^b^*SD* standard deviation

^c^Analyzed by the paired sample *t*-test

^d^An absolute NRS reduction ≥ 1 was considered a clinically important difference

**Table 5:** Symptom intensity and symptom development during hospitalization. Unique patients with moderate to severe symptom intensity (NRS ≥ 4)

Symptom	Sample	Mean NRS ^a^ score			SD ^b^	*p*-value ^c^
		Admission	Discharge	Difference ^d^		
Average pain	98	5.78	3.65	2.12	2.53	< 0.01
Tiredness	146	6.42	4.82	1.61	2.21	< 0.01
Drowsiness	132	6.25	4.64	1.61	2.51	< 0.01
Nausea	47	5.79	2.96	2.83	2.75	< 0.01
Appetite	105	6.61	4.78	1.83	2.82	< 0.01
Shortness of breath	78	5.83	4.03	1.81	2.51	< 0.01
Depression	82	5.96	4.32	1.66	2.39	< 0.01
Anxiety	68	5.78	3.94	1.84	2.61	< 0.01
Well-being	107	5.81	3.74	2.08	1.98	< 0.01
Constipation	67	6.54	2.82	3.72	2.58	< 0.01
Sleep	101	5.99	3.77	2.22	2.85	< 0.01
Worst pain	118	6.65	4.39	2.26	2.86	< 0.01

^a^*NRS* numeric rating scale

^b^*SD* standard deviation

^c^Analyzed by the paired sample *t*-test

^d^An absolute NRS reduction ≥ 1 was considered a clinically important difference

Patients included in the “integrated care pathway” reported higher worst pain intensities at admission compared to patients included in the “palliative care pathway” (mean SI 5.7 vs. 4.7, respectively, *p* = 0.03). For all other symptoms, there were no statistically significant differences in SIs at admission for patients included in the two respective care pathways. Furthermore, drowsiness improved more during hospitalization for patients included in the “integrated care pathway” (mean 1.7 points vs. 0.6 points (NRS 0–10), respectively, *p* = 0.03). For all other symptoms, there were no statistically significant differences in symptom development during the hospital stay for patients included in the two respective care pathways.

## Discussion

The study provided a comprehensive description of interventions during hospitalization at an APCU. The evaluated patients experienced relief for all assessed symptoms, with the larger effect sizes for patients with moderate and severe SIs. Patients receiving systemic cancer therapy reported symptom relief similar to patients not on systemic cancer therapy. Most patients received opioids at admission, and the majority needed opioid dose adjustments during hospitalization.

Patient demographics in the current study are comparable to reports from other APCUs [20, 31]. Almost 40% of the admitted patients were included in the “integrated care pathway,” an approach in line with recommendations from stakeholders and the World Health Organization [12, 32].

### Appraisal of methods

All consecutive admissions to an APCU during 1 year were recorded, and the study provided an unselected registration of interventions applied during hospitalization. Thus, the interventions reflect daily practice.

Symptom development was registered for only a fraction of the admissions and patients with severely reduced cognitive or physical function were not included, making the results prone to selection bias. In addition, the study provides no inference of causality between interventions and symptom relief. Furthermore, the study-related organizational focus on systematic symptom assessment may have resulted in an overestimation of daily practice symptom relief.

The single-center, one-group study design opens for systematic errors [33]. Local organizational structures and the inclusion of few patients with hematological, gynecological, and pulmonary malignancies limit the study’s generalizability.

Almost three-quarters of the patients had comorbidities. The number and severity of comorbidities were not specified, and the reported symptoms may have been caused by conditions other than the cancer or the cancer treatment [34]. The 11-point numeric rating scale provides information on SI. Sometimes a more comprehensive assessment may be needed, e.g. when evaluating symptoms like depression and anxiety [35]. Additionally, important palliative care outcomes like patient and family satisfaction were not assessed.

### Interventions

The majority of the admitted patients underwent diagnostic imaging, almost 40% received antibiotics, and two-thirds needed opioid dose adjustments to achieve pain control. In addition, many patients received other medical interventions, such as fluid therapy, nutrition, and blood transfusions. Furthermore, a considerable number were treated with radiotherapy, radiological interventions, or surgery. The abovementioned, and the high number of emergency admissions, emphasizes the patient population’s needs and demands for urgent hospital care, or emergency palliative care.

Only few studies evaluate the diagnostic and therapeutic interventions used at an APCU. A study of 42 patients admitted to the M.D. Anderson Cancer Center reported comparable use of medications for symptom relief, but more use of interventional procedures [36]. The study design makes comparison difficult and inferring whether our practice represents overtreatment or undertreatment even more so. Still, a critical focus on potentially non-beneficial procedures at the end of life, especially the use of diagnostic imaging and treatment of infections, is necessary [25].

Although participants of the MDT followed up approximately 40% of the patients, involvement from the entire team was less frequent. One might argue that the relatively limited use of the MDT represents undertreatment [32]. On the other hand, most of the patients were prior to admission APCU outpatients, already introduced to the MDT, and not necessarily in need of its attention during the hospital stay. Despite a lack of studies exploring its impact in a controlled framework, the MDT is considered a cornerstone in palliative care. In our study, a quantification of the effect of the MDT on symptom relief was not possible.

### Symptom development

The patient-reported symptom burden at admission was comparable to previous reports from palliative care units [2, 19, 20]. Moreover, the reductions in symptom scores were in line with other studies exploring symptom development during hospitalization [19–21]. Symptoms like tiredness, drowsiness, well-being, lack of appetite, and dyspnea may be difficult to alleviate at the end of life [3, 37]. We included patients earlier in the disease trajectory, possibly contributing to the favorable results.

For patients with moderate to severe SIs, large improvements were reported for constipation, nausea, pain, and sleep. With several available drugs for symptom relief, there might be an enhanced focus on these specific symptoms. The improvement in well-being of two points might reflect the comprehensive care offered. Rehydration and treatment of infections may contribute, as well as the general care and family involvement.

Interestingly, the symptom burdens at admission for patients included in the “integrated care pathway” and the “palliative care pathway,” respectively, were equal and worst pain intensity even higher for patients receiving integrated care. In addition, SI reductions were similar for patients included in the two respective care pathways, except for drowsiness, which decreased more for “integrated care pathway” patients. Although some of the assessed symptoms may represent adverse effects of cancer treatment, even patients relatively early in the cancer disease trajectory seemed to benefit from hospitalization at the APCU. This corresponds with previous studies, in which symptom self-reporting during cancer treatment was associated with clinical benefits and increased survival [13, 38].

### Implications and further studies

The study demonstrated reduced SIs for hospitalized patients assessed with PROMs and receiving palliative care. Studies aiming to evaluate the effect of specific interventions may utilize the results for sample size calculations.

Worst pain past 24 h differed significantly from average pain past 24 h. Reducing worst SIs is a treatment goal, and a dynamic comparison of worst and average SIs during hospitalization might be addressed in future research. Additionally, the most intense symptoms may not necessarily be the most bothersome [39]. This aspect also needs more attention.

Finally, the regular daily practice yielded symptom relief. A supplementary decision support system may contribute positively, but further studies are warranted [29, 40].

## Conclusions

The study described the practice and clinically meaningful symptom relief during hospitalization at an APCU. Improvements were similar for patients on systemic cancer therapy and palliative care patients, supporting a benefit of early integration of palliative care into cancer care.

## Data Availability

The corresponding author has full control of all primary data. The dataset generated and/ or analyzed are available from the corresponding author on request.

## Code availability

N/A.
